# Medical Therapy versus Bariatric Surgery in Kidney Transplant Candidates

**DOI:** 10.34067/KID.0000000813

**Published:** 2025-04-14

**Authors:** Beata Bzoma, Anum Iqbal, Tayyab S. Diwan, Aleksandra Kukla

**Affiliations:** 1 Division of Nephrology and Hypertension, Department of Medicine, Mayo Clinic, Rochester, Minnesota; 2 Department of Nephrology, Transplantology and Internal Diseases, Medical University of Gdańsk, Gdańsk, Poland; 3 Department of Surgery and Immunology, Mayo Clinic, Rochester, Minnesota; 4 Von Liebig Transplant Center, Mayo Clinic, Rochester, Minnesota

**Keywords:** kidney transplantation, obesity

## Addressing Obesity in Kidney Transplant Candidates

**Figure 1:**
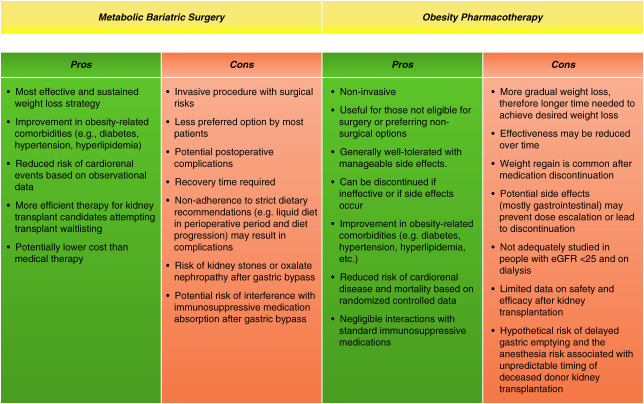
Pros and cons of medical therapy versus bariatric surgery for weight loss in kidney transplant candidates.

Obesity is rising at epidemic proportions among patients with advanced CKD (ACKD), defined as an eGFR <30 ml/min per 1.73 m^2^ and ESKD. Obesity is a significant barrier to accessing kidney transplantation (KT).^1^ Candidates with obesity generally wait longer for the KT or may be denied listing based on individual transplant center criteria.^1^ Patients with a body mass index (BMI) over 40 are almost universally denied because of the independent association of obesity with increased perioperative complications and potentially long-term cardiometabolic risks. Effective and safe obesity treatment can facilitate KT and potentially improve cardiometabolic health. Both medical and surgical weight loss options can be considered alternative or complementary approaches for KT candidates (Figure 1). This article discusses medical and surgical treatments for obesity in KT candidates seeking to lose weight to qualify for KT and/or improve obesity-related comorbidities.

## Weight Loss Pharmacotherapy

The emergence of highly effective weight loss pharmacotherapy, such as incretin mimetics, has led to renewed interest in using these agents for weight loss in patients with ACKD. Incretin mimetics, including agonists of glucagon-like peptide-1 receptor (GLP-1 RAs) and glucose-dependent insulinotropic polypeptide receptor (GIP RA), work by suppressing appetite through central nervous system pathways and slowing gastric emptying to prolong fullness. These agents increase insulin secretion and inhibit glucagon release, which helps stabilize blood glucose levels and indirectly supports weight management.^2^ In the general population, clinical trials have shown that individuals taking 2.4 mg of semaglutide (a GLP-1 RA) experienced an average total weight loss of 14.9% at 68 weeks, while those taking 15 mg of tirzepatide (a GLP-1/GIP RA) achieved an average weight loss of 20.9% at 72 weeks.^2^ Data on the safety of GLP-1 RAs and GLP-1/GIP RA in ACKD are limited. Among these two best-in-class agents, semaglutide has more tolerability and safety data in patients with ACKD than tirzepatide. The effect of semaglutide versus placebo on the progression of renal impairment in people with type 2 diabetes and CKD trial tested the effect of semaglutide on kidney outcomes in 3533 people with type 2 diabetes (T2D) and CKD (eGFR of 25–75 ml/min per 1.73 m^2^), including 400 individuals (11%) with eGFR <30 ml/min per 1.73 m^2^. The maximum dose of semaglutide used in the FLOW trial was 1.0 mg, and the average weight loss was 4.10 kg.^3^ The tirzepatide versus insulin glargine in type 2 diabetes and increased cardiovascular risk (SURPASS-4 trial) enrolled 18% of patients with eGFR <60 ml/min per 1.73 m^2^, among whom only 22 patients (1%) had eGFR <30 ml/min per 1.73 m^2^.^4^ Both studies demonstrated kidney and cardiovascular benefits of incretin-based therapies. However, they did not report subgroup analyses based on GFR and did not include patients with ESKD, who are more likely to be waitlisted for KT. Retrospective studies with GLP-1 RAs (or GLP-1/GIP RA) in ESKD are scarce. Long and colleagues reported acceptable tolerability in patients with ACKD (approximately 9% of patients discontinued semaglutide due to the gastrointestinal [GI] side effects) and safety in this population.^5^ Most patients in this analysis were treated for T2D. Therefore, semaglutide was prescribed in lower doses than what is approved for the treatment of obesity. Patients achieved modest weight loss (average of 5 kg). Retrospective data also support cardiovascular benefits and no significant harm related to GLP-1 RAs use in patients starting dialysis.^6^ Hypothetically, however, the population with ACKD may have less tolerability than individuals with normal kidney function because of a higher incidence of GI symptoms related to renal failure, dialysis, and comorbidities (*e.g*., diabetic gastroparesis). Moreover, patients not yet on dialysis can also hypothetically experience AKI if dehydration occurs because of nausea and vomiting. The lack of prospective studies with gradual dose escalation to the maximum tolerated dose of GLP-1 RAs (and GLP-1/GIP RA) limits our understanding of tolerability and expected weight loss in this high-risk population. Therefore, further prospective studies are needed to inform the practice.

Weight regain after discontinuation of therapy, documented in the general population,^2^ is also likely to occur in patients with ACKD. Therefore, patients should be advised that the treatment will likely need to continue for weight maintenance after the transplant. Although KT recipients have not been included in randomized controlled trials with GLP-1 RAs (or GLP-1/GIP RA), emerging retrospective data do not show significant issues with transplant medication absorption, increased rejection risk, or adverse effects on allograft survival and, on the contrary, suggest potential cardiometabolic and kidney allograft benefits.^7^

## Metabolic Bariatric Surgery

Metabolic bariatric surgery (MBS) can be considered in people with a BMI >40 and those with a BMI >35 who have severe obesity-related comorbidities such as T2D, according to the current recommendations.^2^ MBS typically results in >20% body weight loss and significantly improves glycemic control (often leading to T2D remission), cardiovascular outcomes, and life expectancy in the general population.^2^ In KT candidates, studies report approximately 17% weight loss at 6 months.^8^ Weight loss can be achieved more rapidly with MBS than with medical therapy, with over 50% of total weight loss occurring within the first 2 months.^8^ Therefore, MBS may be more effective in achieving KT listing (shorter time to waitlisting and a higher likelihood of receiving KT) than the nonsurgical approach, although no randomized controlled trials have compared these two modalities. Significant improvement and even reversal of comorbidities (such as hypertension and T2D) may be expected post-MBS, leading to the overall improvement in cardiometabolic health, with as many as 30% of patients with ACKD discontinuing antihyperglycemic agents.^8^ Sleeve gastrectomy may be preferred over Roux-en-Y gastric bypass because of fewer surgical complications,^9^ no risk of kidney stones or oxalate nephropathy, and no significant interference with medication absorption. However, gastric bypass can also be performed safely in KT candidates, depending on the experience of the bariatric center, especially when there are contraindications to sleeve gastrectomy (such as GI reflux or hiatal hernia) or when more significant weight loss is desired.^9^ Regardless, thorough monitoring post-MBS is required for the optimal management of rapidly changing obesity-related comorbidities and management of potential surgery-related complications.^10^ Weight regain may also occur after MBS but to a lesser degree than after the discontinuation of incretin-based therapies.^2^

## Conclusion

Incretin-based therapies are promising noninvasive options for weight loss in KT candidates, although data on their effectiveness in ACKD are limited. Long-term use may lead to weight plateaus, and stopping treatment can cause weight regain. MBS should be prioritized in patients with larger weight loss required and those with severe obesity-related comorbidities, such as T2D or intolerance to GLP-1 RA (GIP RA). MBS may be less beneficial for older individuals (*e.g*., older than 70 years), patients with higher surgical risks, patients expected to remain on dialysis for a longer period, or patients at high risk for sarcopenia/frailty. Incretin-based therapies can be effectively used as an alternative to bariatric surgery or in combination with it. These agents can serve as a bridge to surgery or help maintain weight and manage weight regain. Individuals with ACKD undergoing supervised weight loss, whether surgical or medical, should be monitored to ensure they receive appropriate nutrition. Efforts should focus on “healthy weight loss,” emphasizing the preservation of muscle mass. More studies are needed to understand the long-term pretransplant medical and surgical weight loss therapies on kidney and patient outcomes post-transplant.
